# CFD simulation of the effect of particle size on the nanofluids convective heat transfer in the developed region in a circular tube

**DOI:** 10.1186/2193-1801-2-192

**Published:** 2013-04-30

**Authors:** Reza Davarnejad, Sara Barati, Maryam Kooshki

**Affiliations:** Department of Chemical Engineering, Faculty of Engineering, Arak University, Arak, 38156-8-8349 Iran; Department of Chemistry, Faculty of Science, Arak University, Arak, 38156-8-8349 Iran

**Keywords:** Nanofluid, Heat transfer, Simulation, Laminar flow

## Abstract

The CFD simulation of heat transfer characteristics of a nanofluid in a circular tube under constant heat flux was considered using Fluent software (version 6.3.26) in the laminar flow. Al_2_O_3_ nanoparticles in water with concentrations of 0.5%, 1.0%, 1.5%, 2% and 2.5% were used in this simulation. All of the thermo-physical properties of nanofluids were assumed to be temperature independent. Two particle sizes with average size of 20 and 50 nm were used in this research. It was concluded that heat transfer coefficient increased by increasing the Reynolds number and the concentration of nanoparticles. The maximum convective heat transfer coefficient was observed at the highest concentration of nano-particles in water (2.5%). Furthermore, the two nanofluids showed higher heat transfer than the base fluid (water) although the nanofluid with particles size of 20 nm had the highest heat transfer coefficient.

## Introduction

Fluid heating and cooling play the significant roles in a lot of industrial processes such as power stations, production processes, transportation and electronics. Most of the methods for heat transfer are based on the structure variation, vibration of the heated surface, injection or suction of fluid and applying electrical or magnetic fields ( Ahuja 1975 ; Bergles & Webb 1970 ; Bergles 1973 ). These techniques meet a great enhancement in heat flux. Heat transfer in the traditional fluids such as water, ethyleneglycol and oil inherently has low thermal conductivity compared with the metals and metal oxides. Therefore, fluids with suspended solid particles are expected to have better heat transfer properties ( Eastman et al. 2001 ). Due to the associated technological problems, the majority of studies on heat transfer of suspension of metal oxides in fluids were limited to suspensions with millimeter or micron-sized particles. The large particles may cause severe problems in the heat transfer equipments. In particular, large particles quickly tend to settle out. So, pressure drop can occur in the micro-channels ( Khanafer et al. 2003 ). Furthermore, the abrasive actions of the particles cause the erosion phenomenon in pipe lines. Small particles and their little volume fractions prevent particles clogging and pressure drop increment in the nanofluids ( Khanafer et al. 2003 ; Zhou 2004 ). Moreover, large surface area of nanoparticles increases the stability and reduces the sedimentation of nanoparticles. A more dramatic improvement in heat transfer efficiency is expected as a result of the particle size reduction in a suspension because heat transfer takes place at the particles surface ( ZeinaliHeris et al. 2007 ).

Choi employed the particles in nanometer dimensions as a suspended solution ( Choi & Eastman 1995 ). He showed that the nanofluid thermal conductivity considerably increased. Lee et al. showed that the suspension of 4.0% with 35 nm Cuo particles in ethylene glycol had 20% increment in the thermal conductivity ( Lee et al. 1999 ). Choi et al. observed 60% enhancement in the thermal conductivity of engine oil with 1.0% carbon nanotube ( Choi et al. 2001 ). Das et al. investigated the temperature dependency of thermal conductivity in the nanofluids ( Das et al. 2003 ). It was observed that a 2–4 fold increase in the thermal conductivity of nanofluid can take place over a temperature range of 21–51°C.

According to the literature, alumina and copper oxide are the most ordinary and cheap nanoparticles which are used in the applied processes ( Rezaee & Tayebi 2010 ). Xuan and Li experimentally studied the convective heat transfer and friction coefficient for the nanofluid in both laminar and turbulent flows ( Xuan & Li 2000 ; Xuan & Li 2003 ). According to these researches, the flow velocity and volume fraction of nanoparticles affected the heat transfer coefficient. Wen and Ding investigated the convective heat transfer characteristics in Al_2_O_3_-water nanofluid along a tube ( Wen & Ding 2004 ). It was observed that heat transfer increased by increasing the Reynolds number and volumetric ratio of particles.

Abu-Nada investigated the effects of variable viscosity and thermal conductivity of a nanofluid (Al_2_O_3_-water) on the natural convective heat transfer ( Abu-Nada 2009 ). Sharma et al. experimentally studied the convective heat transfer coefficient and pressure drop in the transient region for Al_2_O_3_-water nanofluid under a constant heat flux ( Sharma et al. 2009 ). They found that convective heat transfer increased by adding Al_2_O_3_ nanoparticles in water.

Mirmasoumi et al. numerically studied the convective heat transfer in a fully developed flow for Al_2_O_3_-water nanofluid ( Mirmasoumi & Behzadmehr 2008 ). They applied two-phase mixture model in their simulation. They found that the convective heat transfer coefficient significantly increased by decreasing the nanoparticles mean diameter. Since the theoretical models such as Maxwell and Hamilton-Crosser ( Keblinski et al. 2002 ; Xue & Xu 2005 ; Eastman et al. 2001 ) predict the thermal conductivity of nanofluids, the mechanisms of thermal conductivity enhancement in the nanofluids should be studied.

Moraveji et al. simulated water-Al_2_O_3_ nanofluid through a tube under constant heat flux ( Moraveji et al. 2011 ). They found that the heat transfer coefficient increased by increasing the nanoparticle concentration and Reynolds number. Furthermore, the heat transfer coefficient increased by decreasing particle diameter.

In this research, the convective heat transfer in the developed region of the tube flow containing water and Al_2_O_3_ nanofluid under constant heat flux was simulated using the Computational Fluid Dynamics (CFD) tools. Al_2_O_3_ nanoparticles with two average diameters of 20 nm and 50 nm were dispersed in water. The nanofluids with five different Al_2_O_3_ nanoparticle concentrations (0.2%, 1.0%, 1.5%, 2.0% and 2.5% volume fraction) were used. Effects of nanoparticle size and their concentrations on the convective heat transfer coefficient were also investigated.

## Mathematical modeling

The nanofluid as a single phase fluid with different physical properties (density, thermal conductivity and viscosity) was applied for the simulation. Heat transfer and flow are considered by the continuity, momentum and energy equations (Izadi et al. 2009):

Continuity equation:1

Momentum equation:2

Energy equation:3

The physical properties for above equation can be obtained ( Ghasemi & Aminossadati 2009 ):4

The effective heat capacity is calculated by ( Ghasemi & Aminossadati 2011 ):5

The viscosities of nanofluid (with the average particle size of 20 and 50 nm) can be predicted by Einstein’s equation:6

In this research, the single-phase approach was applied. Solid particles with less than 100 nm diameter were spotted in the single-phase approach. Moreover, some necessary data for the thermal conductivities determination for various concentrations of nanofluid were obtained from the literature ( ZeinaliHeris et al. 2007 ). Yu and Choi’s correlation ( Yu & Choi 2003 ; Trisaksri & Wongwises 2007 ) was applied for the nanofluid effective thermal conductivity determination:7

Where, β is the ratio of the nano-layer thickness to the original particle radius (β = 0.1) ( Yu & Choi 2003 ). The rheological and physical properties of the nanofluid were calculated at the average bulk temperature.

**Figure 1:**
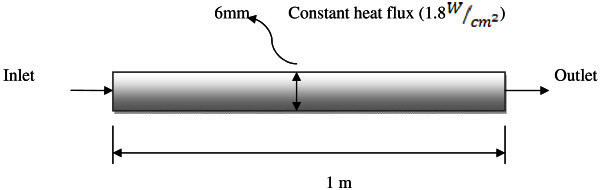
**Pipe numerical domain.**

As shown in Figure 1, a two dimensional pipe (with 1 m length and 6 mm inner diameter) was spotted in our simulation. The single phase approach was used for nanofluid simulation and effect of nanoparticle concentration on the convective heat transfer coefficient was investigated in the various Reynolds numbers (700 < Re < 2050).There were 20 meshes in the radial direction with a size ratio of 1 from the center to the wall. The constant heat flux of 1.8 (W/cm^2^) as a boundary condition at the pipe wall was applied.

### CFD simulation procedure

**Figure 2:**
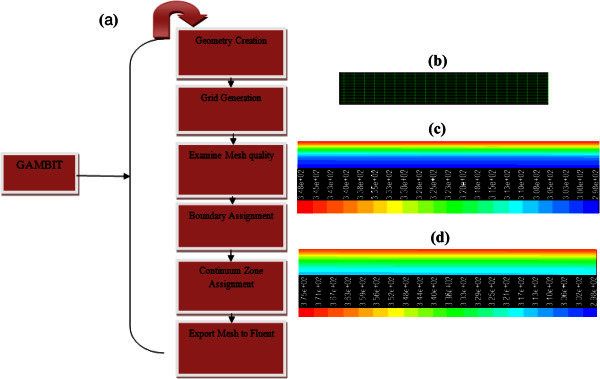
**Temperature map at the end part of the pipe for the nanofluid (20 nm & 2.5%).** (**a**). GAMBIT steps during meshes generation. (**b**). Meshes generation. (**c**). U = 0.1664 (m/s). (**d**). U = 0.0706 (m/s).

The geometry and the gird were generated using GAMBIT. The GAMBIT is an integrated preprocessor for CFD analysis. The sequences of GAMBIT steps are shown in Figure 2a. There are 20 meshes in the radial direction with a size ratio of 1 from the center to the wall. Further, there are 1000 meshes in the horizontal direction with an average size. The sequences of GAMBIT steps are shown in Figure 2a. The physical boundary conditions for the geometry are defined as inlet, outlet and wall of the pipe. The continuum was the fluid. Then, the mesh file was successfully conducted into the Fluent (version 6.3.26). For single phase approach, solid particles with diameter less than 100 nm were spotted. Therefore, single phase approach was adopted for nanofluid modeling ( Moraveji et al. 2011 ). The fluid was entered the pipe with a constant velocity in each run. The initial temperature of fluid (at t = 0) was 25°C. The Symmetric option was chosen in the software. The constant heat flux of 1.8 (W/cm^2^) as a boundary condition at the pipe wall was applied.

## Results and discussion

The local heat transfer coefficient and local Nusselt number were calculated using the following equations:89

Where, D, *q*, *h*_*nf*_, k, *T*_*w*_ and *T*_*nf*_ are pipe diameter, heat flux, nanofluid heat transfer coefficient, thermal conductivity of the fluid, tube wall temperature and nanofluid temperature, respectively.

Figure 2b demonstrates the meshes generation in this research. Figure 2c and d show temperature distribution at the end of pipe for velocity of 0.0706 and0.1664 (m/s) (or Re = 420 and 991.6) for nanofluid (with the particle diameter of 20 nm and volume fraction of 2.5). The nanofluid was heated by the pipe wall and its temperature increased along the pipe. The temperature of nanofluid along the pipe at Reynolds number of 420 varied sharper than that of Reynolds number of 991.6.Its reason was due to magnifying the heat transfer coefficient ( He et al. 2009 ).

**Figure 3:**
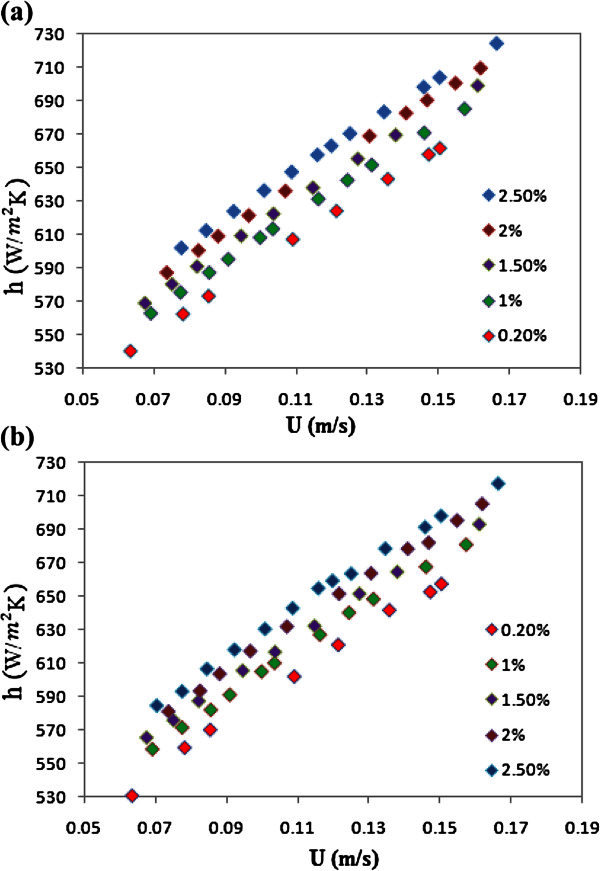
**(a). Heat transfer coefficient versus velocity for the nanofluid with particle size of 20 nm.** (**b**). Heat transfer coefficient velocity for the nanofluid with particle size of 50 nm.

Figure 3a and b show heat transfer coefficient in the tube versus velocity for various concentrations of nanoparticles (with diameters of 20 and 50 nm). According to these figures, heat transfer coefficients increased by increasing the volume fraction and velocity.

**Figure 4:**
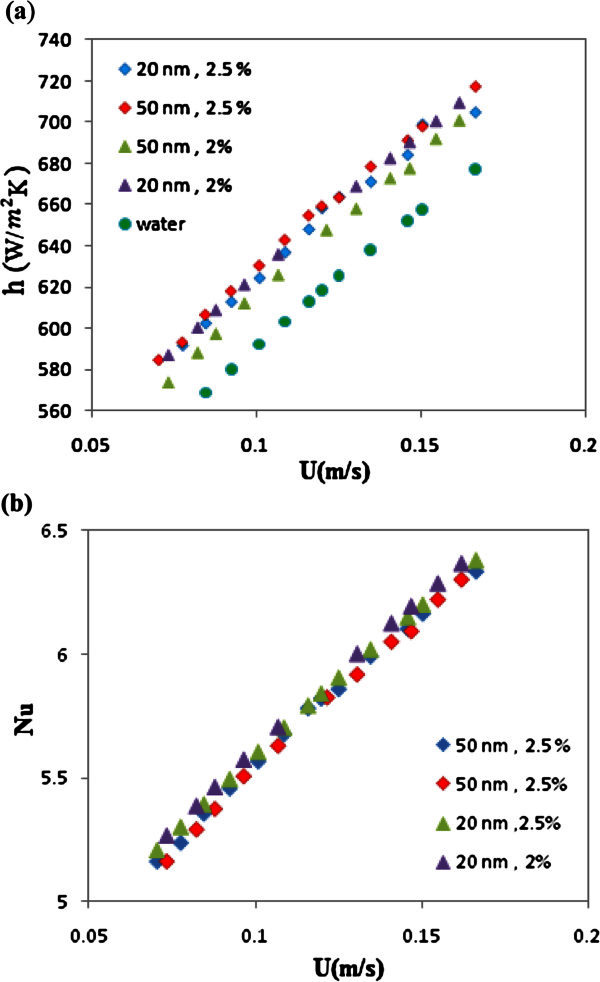
**(a). Effect of particle size on the heat transfer coefficient in the various velocities.** (**b**). Effect of particle size on the Nusselt number in the various velocities.

Figure 4a and b show heat transfer coefficients (*h*) and Nusselt (*Nu*) number in the tube versus velocity at particle concentrations of 2.5 and 2% for both particle sizes. As shown in these figures, heat transfer coefficients increased by increasing velocity and decreasing particle diameter. Furthermore, the nanofluid with average particle size of 20 nm showed the maximum heat transfer ( Anoop et al. 2009 ).

**Figure 5:**
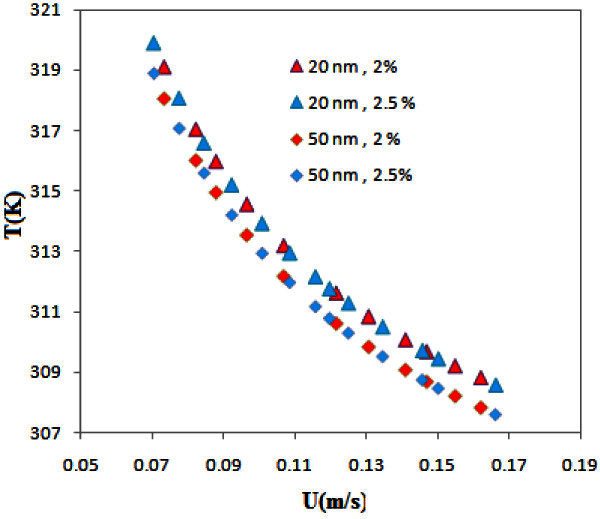
**Temperature of nanofluid versus velocity for various concentrations of nanoparticles.**

Figure 5 shows nanofluid temperature versus velocity for particles of 20 and 50 nm (for volume fractions of 2.5 and 2). As show in this figure, the nanofluid temperature decreased by increasing the particle size although a change in the volume fraction had no significant effect on the nanofluid temperature. The experimental results investigated by Anoop et al. ( 2009 ) were in very good agreement with our theoretical data and supported them.

## Conclusions

In this article, the heat transfer coefficient in the developed region of pipe flow containing Al_2_O_3_-water nanofluid during the constant heat flux was simulated using CFD. The focal point of investigation was to evaluate the effect of particle size on convective heat transfer characteristics in the developed region of the tube flow containing water-Al_2_O_3_ nanofluid. It was observed that both nanofluids (with 20 and 50 nm particles size) showed higher heat transfer characteristics than that of the base fluid (water). Furthermore, the nanofluid with 20 nm particles size showed the highest heat transfer coefficient. The average heat transfer coefficient and Nusselt number increased by increasing the particle concentration and flow rate. The average temperature of nanofluid decreased by increasing the particles size.

## Nomenclature

D diameter of the tube [m]

k thermal conductivity [W/m.K]

Nu Nusselt number

Re Reynolds number

T Temperature [K]

C specific heat (*J/*_*kg.K*_)

q heat flux [W/]

x distance a long axis [m]

## Subscripts

w wall

nf nanofluid

In inlet

p particle

Out outlet

bf base fluid

f fluid

## Greek letters

*μ* viscosity (Pa.s)

ф volume fraction
